# Endovascular treatment for acute basilar artery occlusion via persistent primitive hypoglossal artery: A case report

**DOI:** 10.1097/MD.0000000000027998

**Published:** 2021-12-03

**Authors:** Jung Soo Park, Byoung-Soo Shin, Hyun Goo Kang

**Affiliations:** a Department of Neurosurgery, Jeonbuk National University Medical School and Hospital, Jeonju, South Korea; b Research Institute of Clinical Medicine of Jeonbuk National University - Biomedical Research Institute of Jeonbuk National University Hospital, Jeonju, South Korea.; c Department of Neurology, Jeonbuk National University Medical School and Hospital, Jeonju, South Korea.

**Keywords:** basilar artery, persistent primitive hypoglossal artery, thrombectomy

## Abstract

**Introduction:**

Although their effectiveness and safety have not yet been established, endovascular treatments have recently been applied in the treatment of acute basilar artery occlusion. If not identified, persistent primitive hypoglossal artery, a rare variant of the posterior circulation, could be a barrier to the successful treatment of basilar artery occlusion.

**Patient concerns:**

An 83-year-old woman, who had been undergoing treatment for hypertension for 20 years, visited our hospital 3 hours after the onset of acute unresponsive mental deterioration. The patient was unresponsive to painful stimuli, and the pupils were equal and miotic.

**Diagnosis:**

Brain computed tomography angiography confirmed complete occlusion of the distal basilar artery and revealed a dilated branch arising from the right internal carotid artery at the C2 vertebral level.

**Interventions:**

Endovascular thrombectomy was performed directly via the right femoral artery. Complete recanalization was achieved via manual aspiration thrombectomy.

**Outcomes:**

Brain magnetic resonance imaging revealed multifocal cerebral infarctions in the bilateral thalamus, midbrain, and cerebellar vermis. The patient's neurological symptoms gradually improved.

**Conclusions:**

This is a rare case of basilar artery occlusion that was successfully treated with mechanical thrombectomy through persistent primitive hypoglossal artery. It is important to consider the potential clinical implications of this rare vascular variant.

## 1 Introduction

Although several recent randomized controlled trials have prompted the use of endovascular treatment (EVT) in patients with acute large vessel occlusion, evidence of its efficacy and safety in the treatment of basilar artery (BA) occlusion is lacking.^[1,2]^ Persistent primitive hypoglossal artery (PPHA), the second most common persistent carotid-vertebrobasilar anastomosis, is a rare variant of posterior cerebral circulation.^[3]^ The persistence of this fetal anastomosis may be associated with hypoplasia of the ipsilateral vertebral artery (VA), as well as intracranial vascular anomalies such as aneurysms.^[4,5]^

Here, we describe an interesting and rare case of a patient with BA occlusion with PPHA, who was successfully treated with EVT through this persistent fetal anastomosis route.

## 2 Case presentation

**Figure 1:**
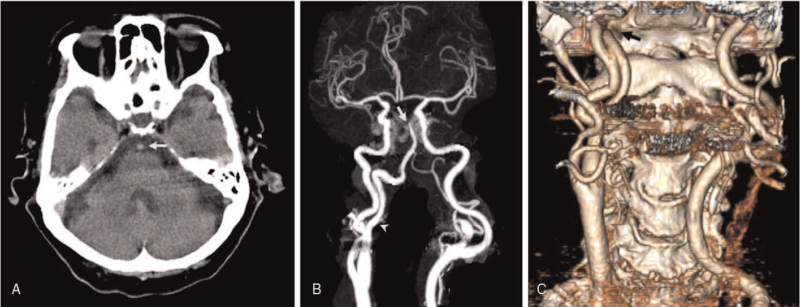
(A) Non-enhanced head computed tomography (CT) reveals a hyperdense basilar artery (white arrow). (B) CT angiography (CTA) reveals complete occlusion of the distal basilar artery (white arrow) and dilated branch arising from the right internal carotid artery (white arrowhead). (C) Three-dimensional reconstruction imaging of CTA shows a dilated branch arising at the C2 level, extending posteriorly and passing through the enlarged hypoglossal canal (black arrow).

An 83-year-old woman, who had been undergoing treatment for hypertension for 20 years, presented to our emergency department 3 hours after the onset of acute unresponsive mental deterioration. The patient was unresponsive to painful stimuli, and the pupils were equal and miotic. Her initial National Institutes of Health Stroke Scale scores were 20. Laboratory tests and electrocardiogram findings revealed no abnormalities. The patient was emergently intubated, and non-enhanced brain computed tomography (CT) and CT angiography were performed sequentially. Brain CT revealed a hyperdense BA without any focal hypodense lesions (Fig. 1A). Subsequent computed tomography angiography confirmed complete occlusion of the distal BA and revealed a dilated branch arising from the right internal carotid artery (ICA) at the C2 vertebral level. This unusual branch predominantly supplying the BA extended posteriorly and passed through the enlarged hypoglossal canal (Fig. 1B, C). These findings are consistent with the descriptions of PPHA.

**Figure 2:**
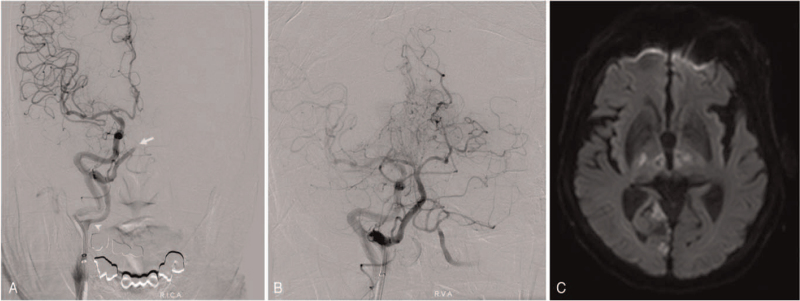
(A) Distal subtraction angiography shows the distal basilar artery occlusion (white arrow). The origin of the persistent primitive hypoglossal artery is at the proximal cervical segment of the internal carotid artery (white arrowhead). (B) Postprocedural angiography shows complete recanalization of the occluded basilar artery. (C) Postprocedural magnetic resonance imaging of the brain reveals multifocal cerebral infarctions in the bilateral thalamus, midbrain, and cerebellar vermis.

EVT was planned through the PPHA as the left VA was hypoplastic. After right femoral artery puncture, a 100-cm 8F guide catheter (Guider Softip; Stryker, Natrick, MA, USA) was advanced to the right cervical ICA just above the ICA bifurcation (Fig. 2A). Complete recanalization was achieved by manual aspiration thrombectomy with a 4MAX Penumbra reperfusion catheter (Penumbra, Alameda, CA), navigated triaxially over a Rebar 18 microcatheter (EV3, Irivine, CA) with a Synchro 0.014-inch guidewire (Stryker, Freemont, CA) (Fig. 2B).

Postprocedural magnetic resonance imaging of the brain revealed multifocal cerebral infarctions in the bilateral thalamus, midbrain, and cerebellar vermis (Fig. 2C). The patient's neurological symptoms gradually improved. After 1 month of rehabilitation, the patient's level of consciousness improved to the “drowsy” state, and although she showed mild dysarthria, she was able to communicate.

## 3 Discussion

At the 3 to 4 mm embryonic stage, the forebrain is supplied by the carotid system. Two parallel longitudinal arteries run along the surface of the hindbrain, which eventually fuse to form the BA. During this embryonic stage, various anastomotic channels are present between the carotid and vertebrobasilar systems. By the 7 to 12 mm embryonic stage, these channels start to regress and disappear when the embryo is approximately 12 to 14 mm long.^[6]^ In rare cases, some of the channels fail to regress and are retained in the adult cerebral circulation. These retained channels may be called persistent trigeminal, otic, hypoglossal, or proatlantal intersegmental arteries, depending on their anatomic location.

The PPHA is the second most common carotid-vertebrobasilar anastomosis (0.03%–0.09%) after the primitive trigeminal artery (0.2%).^[7]^ Arnould et al have reported the following anatomic and angiographic criteria for identifying PPHA: (i) it arises from the extracranial cervical part of the ICA; (ii) it enters the skull via the hypoglossal canal; and (iii) the BA originates from it.^[8]^

In most cases, PPHA may be detected as an incidental finding during cerebral angiography, as it is usually asymptomatic. However, in rare cases, it may cause neurological symptoms, such as glossopharyngeal neuralgia, or may be accompanied by other cerebrovascular diseases.^[9]^ When found in conjunction with a cerebrovascular disease, PPHA is mostly associated with aneurysms. It has previously been suggested that PPHA may be associated with the anomalous structure of the vessel wall and expose the basilar trunk to unusual excessive hemodynamic stress, thus predisposing to the formation of aneurysms.^[10,11]^ In addition to these hemodynamic characteristics, although a rare anatomical variation, the anatomical characteristics of PPHA are clinically significant if carotid endarterectomy or skull base surgery are planned. Additionally, in the case of ischemic stroke, the anomalous anatomical patterns observed in patients with PPHA can result in complex neurological presentations. Because most patients with PPHA present with bilateral hypoplastic VA or a hypoplastic contralateral VA and an absent ipsilateral VA, the majority of the blood supply to the posterior circulation travels via the PPHA. Therefore, in ischemic stroke patients with PPHA, regardless of the etiology being cardioembolic or atherosclerotic, both the anterior and posterior circulations are often involved.^[12,13]^ To the best of our knowledge, there are several cases of ischemic stroke in the posterior circulation with PPHA, but only two cases of EVT through PPHA in patients with BA occlusion have been reported.^[14,15]^

Considering the recent trend of gradually expanding the scope of EVT in the treatment of acute ischemic stroke, clinicians should be aware of these rare anatomical variations in advance. Since the prognosis of patients undergoing EVT is closely related to early revascularization, understanding of the anatomical features of PPHA could be beneficial for the fast and accurate navigation in rare but embarrassing settings of EVT for neurointerventionists.

## Acknowledgments

The authors thank Editage (www.editage.co.kr) for English language editing.

## Author contributions

JSP, BSS, and HGK were responsible for the study design. JSP and HGK performed the data collection and analysis. JSP and HGK performed the computational studies and wrote the manuscript. All authors have read and approved the final manuscript.

**Conceptualization:** Jung Soo Park, Hyun Goo Kang.

**Data curation:** Jung Soo Park.

**Formal analysis:** Byoung-Soo Shin, Hyun Goo Kang.

**Funding acquisition:** Jung Soo Park.

**Investigation:** Byoung-Soo Shin, Hyun Goo Kang.

**Methodology:** Jung Soo Park, Byoung-Soo Shin.

**Resources:** Hyun Goo Kang.

**Supervision:** Byoung-Soo Shin, Hyun Goo Kang.

**Validation:** Hyun Goo Kang.

**Visualization:** Hyun Goo Kang.

**Writing – original draft:** Jung Soo Park.

**Writing – review & editing:** Hyun Goo Kang.

## References

[1] Mokin M Sonig A Sivakanthan S. Clinical and procedural predictors of outcomes from the endovascular treatment of posterior circulation strokes. Stroke 2016;47:782–8. 26888533 10.1161/STROKEAHA.115.011598

[2] Singer OC Berkefeld J Nolte CH. Mechanical recanalization in basilar artery occlusion: The ENDOSTROKE study. Ann Neurol 2015;77:415–24. 25516154 10.1002/ana.24336

[3] Hähnel S Hartmann M Jansen O Sartor K. Persistent hypoglossal artery: MRI, MRA and digital subtraction angiography. Neuroradiology 2001;43:767–9. 11594429 10.1007/s002340100566

[4] Hatayama T Yamane K Shima T Okada Y Nishida M. Persistent primitive hypoglossal artery associated with cerebral aneurysm and cervical internal carotid artery stenosis—case report. Neurol Med Chir (Tokyo) 1999;39:372–5. 10481441 10.2176/nmc.39.372

[5] Huynh-Le P Matsushima T Muratani H Hikita T Hirokawa E. Persistent primitive hypoglossal artery associated with proximal posterior inferior cerebellar artery aneurysm. Surg Neurol 2004;62:546–51. discussion 551. 15576127 10.1016/j.surneu.2004.03.018

[6] Padget DH. Designation of the embryonic intersegmental arteries in reference to the vertebral artery and subclavian stem. Anat Rec 1954;119:349–56. 13197795 10.1002/ar.1091190306

[7] Srinivas MR Vedaraju KS Manjappa BH Nagaraj BR. Persistent primitive hypoglossal artery (PPHA)—a rare anomaly with literature review. J Clin Diagn Res 2016;10: TD:13-14. 10.7860/JCDR/2016/15556.7116 PMC4740676 26894148

[8] Arnould G Tridon P Laxenaire M Picard L Weber M Gougaud G. The primitive hypoglossal artery. Anatomic and radio-clinical study. Apropos of 2 cases. Rev Neurol (Paris) 1968;118:372–9. 5688566

[9] Bapuraj JR Ojili V Khandelwal N Shanbhogue AK Gupta SK. Basilar artery aneurysm treated with coil embolization via persistent primitive hypoglossal artery. Australas Radiol 2007;51: (suppl): B340–3. 17991102 10.1111/j.1440-1673.2007.01759.x

[10] Sakai K Tanaka Y Tokushige K Tanabe A Kobayashi S. Basilar bifurcation aneurysms associated with persistent primitive hypoglossal artery. Neurosurg Rev 1998;21:290–4. 10068193 10.1007/BF01105788

[11] Yokota N Yokoyama T Ryu H. Aneurysm of persistent primitive hypoglossal artery. Br J Neurosurg 1999;13:608–10. 10715735 10.1080/02688699943178

[12] Sunada I Yamamoto S Matsuoka Y Nishimura S. Endarterectomy for persistent primitive hypoglossal artery—case report. Neurol Med Chir (Tokyo) 1991;31:104–8. 1715037 10.2176/nmc.31.104

[13] Uzawa A Aotsuka A Terano T. Posterior cerebral artery territory infarction associated with persistent primitive hypoglossal artery with internal carotid artery atherosclerosis. Intern Med 2010;49:515–6. 20190498 10.2169/internalmedicine.49.3075

[14] See See AP Baranoski JF Flores BC Ducruet A Albuquerque FC. Basilar stroke from a persistent hypoglossal artery. J Neurointerv Surg 2017;9:e30. 28151414 10.1136/neurintsurg-2016-012859.rep

[15] Voronovich Z Grandhi R Zwagerman NT Jadhav AP Jovin TG. Manual aspiration thrombectomy for basilar infarction in the setting of a persistent primitive hypoglossal artery: Case report and review of the literature. Surg Neurol Int 2014;5:182. 25593766 10.4103/2152-7806.147412 PMC4287913

